# Extending Professional Identity Formation to develop academic faculty for a new medical school

**DOI:** 10.12688/mep.20510.1

**Published:** 2024-07-22

**Authors:** Jeannine Nonaillada, Jason C. Hoffmann, Rob Armstrong Martin

**Affiliations:** 1 NYU Grossman Long Island School of Medicine, Mineola, NY, 11501, USA

**Keywords:** faculty development, faculty engagement, professional identity formation, academic medical center, medical school

## Abstract

Academic health centers have a responsibility to foster professional development approaches and engagement environments for faculty to elevate both knowledge and sense of belonging as medical educators. This new educational methods submission depicts faculty development and engagement initiatives implemented at a single institution that were created and influenced by the psychological framework of Professional Identity Formation. The authors suggest ways that academic medical centers can draw upon the formation of these programs to best serve their faculty for cultivating development and engagement for professional growth.

## Introduction

Academic health centers have a responsibility to foster professional development environments and systematic approaches for faculty to elevate both knowledge and sense of belonging as medical educators. Commonly used to address the questions, ‘who are we?’ and ‘who do we want to become?’, Professional Identity Formation (PIF) is a framework grounded in the psychology of adult development, often used to bridge the gap in physicians and health professionals between who they currently are as care providers and where they want to grow and bridge the gap in other areas ( Sarraf-Yazdi *et al.*, 2024; Sarraf-Yazdi *et al.*, 2021). Under the premise of PIF, an individual’s development is influenced by social-contextual approaches ( Cruess *et al.*, 2019; Jarvis-Selinger *et al.*, 2019). From this lens, PIF can be applied to faculty development and engagement programs to elicit this growth by sharing in communal learning and training, as well as networking with others ( Irby & Hamstra, 2016; Silveira *et al.*, 2023). In contrast with the widely reported use of PIF in the training of medical students and trainees, PIF has only recently begun to gain traction for use by academic faculty in medical schools ( Mahajan *et al.*, 2022; McMains *et al.*, 2023; Mount *et al.*, 2022).

**Figure 1.**
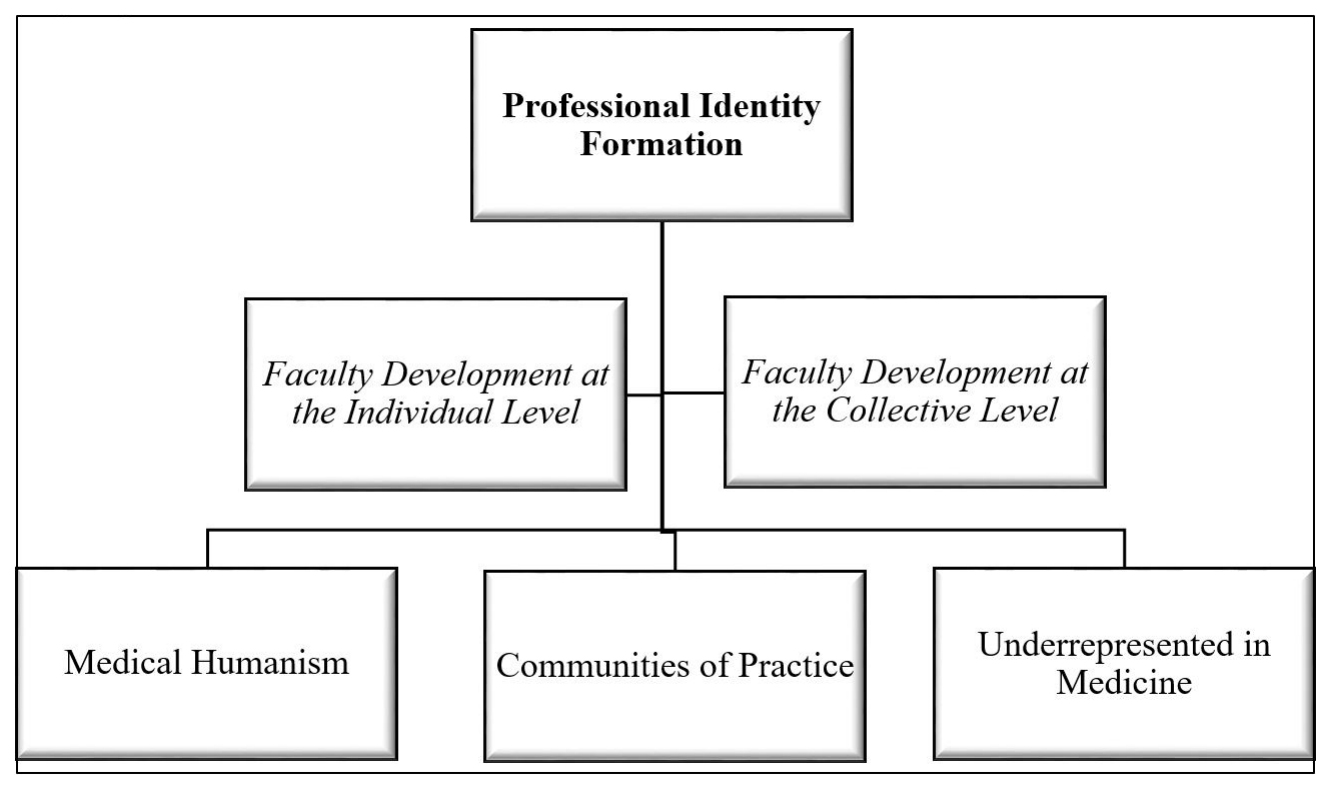
Professional Identity Formation for Faculty Development and Engagement Programming Targeting Structural Characteristics.

In this New Educational Methods submission, we will describe our innovative application of PIF in designing faculty development and engagement programs at our new medical school by leveraging three evidence-based structural characteristics associated with both individual and collective learning ( Mount *et al.*, 2022). These characteristics are: Medical Humanism ( Thibault, 2019), Communities of Practice (CoP) ( Cruess *et al.*, 2018), and Underrepresented in Medicine (URiM) perspective-taking ( Cruess *et al.*, 2015; Spaans *et al.*, 2023), all of which are important for academic medical centers to consider when designing faculty development programs ( Figure 1).

## Method

### Professional Identity Formation as a Faculty Development and Engagement Framework with 3 Structural Characteristics

Theorists of PIF in medical education describe the development of professional identity in medicine ( Cruess *et al.*, 2015), from an early stage in which an individual is primarily motivated to follow rules and be correct when assuming professional roles, through an intermediate stage, when an individual seeks out those to emulate, and finally arriving at a developed stage when the external values of the profession become internal values of the individual, who is also able to control needs, desires, and passions.

Thus, the PIF process moves the individual from existing personal identities (“who you are”) functioning in “legitimate peripheral participation”, toward “full participation” in personal and professional identities of becoming a physician in academic medicine (“who you become”) ( Cruess *et al.*, 2019). We conceptualize this mediating process of socialization to include negotiation, acceptance, compromise and rejection of roles and values, and to rely heavily on a context of social interaction within CoP, acknowledging uniqueness and inclusivity as a substrate of URiM, and embracing internalized values encompassing humanism. It was our deliberate intent to incorporate all of these characteristics into our faculty development and engagement programming.

At the time of this writing, our faculty consist primarily of physicians either with a medical degree (MD) (n = 1033) or an osteopathic degree (DO) (n = 181), both combined equaling 98% of our faculty. The remaining 2% of our faculty hold a research degree (PhD) (n = 31). The duties of faculty vary at the discretion of their department chair, with some having primarily clinical duties, and some having primarily education and research duties. All full-time faculty are required to teach 50 hours per year. Of note, our institution recently transitioned from having faculty at a single hospital to a newly accredited 3-year medical school. As such, it was felt by educational leadership that new programs were necessary to build a working and learning environment with adequate faculty support systems and resources to enhance engagement and efficacy. In doing this, PIF principles were at the foundation of our faculty development and engagement programming, which included:

- A revised longitudinal faculty development program, *Faculty Scholars*, with designated pathways for teaching and research
- A new mentoring program for full time faculty and new special interest groups for junior faculty and faculty identifying as women
- A new coaching program for faculty seeking guidance on educator portfolios or scholarly publications
- Formation of a new faculty council addressing all aspects impacting faculty wellness and benefits

***Faculty scholars: Longitudinal faculty development program*.** Initially formed in 2012, the Faculty Scholars program consisted of didactic classes at a standalone hospital to improve the teaching abilities of our faculty who were serving as clerkship directors and preceptors for 4 ^th^ year clinical campus students from a nearby state university medical school. No formal workplace-sponsored leadership development program for physicians existed at the time of the hospital's designation as a clerkship site affiliated with this state university medical school. The first iteration of Faculty Scholars was centered heavily on didactic, passive delivery of teaching methodology content in the form of lectures, with little opportunities for participant interaction to occur outside of scheduled class time. Although it superficially appeared to meet intended program goals of introducing fundamentals of educator development to our faculty, demonstrating meaningful impact posed a challenge beyond immediate post-session faculty satisfaction feedback. We knew we needed to harness reflective, humanistic experiences of our participants in order to influence PIF in our faculty ( Branch *et al.*, 2017). In 2016, the program was revised to create additional venues for collaborative and experiential learning, including required content posted on virtual team discussion boards, new research content in the curriculum in an effort to produce more scholarly deliverables from graduates, and scheduled meetings with educational and research project mentors serving as role models ( Cruess *et al.*, 2008).

In its current format, the program consists of two pathways: the Educator Pathway of 12 months in duration, emphasizing curriculum development and teaching methods, and the Researcher Pathway of 18 months in duration, focusing on conducting independent research studies ( Kasselman *et al.*, 2022). These pathways encourage faculty to make deliberate choices to enter specific CoP ( Wald *et al.*, 2015). The self-directed element of choosing a named pathway is a first step in identifying a desired CoP, in which members have similar developmental goals. Faculty interested in the program must complete an application and be nominated by their Department Chair, who agrees to allow for the time commitment for these faculty members to participate. Chairs are encouraged to nominate attending faculty physicians who have personal ethnic, cultural, socioeconomic, or gender backgrounds that are URiM, to increase mid-career opportunities for professional inclusion. This is pivotal with identity formation as role modeling who are historically URiM is critical for perceived growth as well as retention ( Spaans *et al.*, 2023; Trevino & Poitevien, 2021). More so, evidence is currently emerging to suggest that some patient outcomes (communication, satisfaction) can be improved where there is cultural, racial, or ethic concordance between patients and their doctors ( Shen *et al.*, 2018; Takeshita *et al.*, 2020). Furthermore, there is rigorous evidence that faculty development programs can increase engagement of members of underrepresented minorities ( Steinert *et al.*, 2019). In particular, engaging faculty input on how to improve institutional climate regarding minority faculty advancement plays a significant role in faculty’s’ perception of the institution, and is suggested to decrease the likelihood that minority member faculty will leave academia prematurely, and abandon scholarly identity formation ( Rodriguez *et al.*, 2014).

***Faculty mentoring programs*.** Mentoring among physicians and health professionals is a crucial part of engagement, retention and satisfaction ( Chua *et al.*, 2020; Huggett *et al.*, 2020). In recognition of this, we have multifaceted programmatic initiatives for mentoring that faculty participate in. All faculty at the Assistant Professor level on the Educator or Scholar tracks, where productivity in teaching and/or research is required in addition to clinical duties, must have a mentor. This mentor meets with them three to four times a year, and mutual collaboration is documented on an Annual Mentoring Letter. This document serves as a tangible, visible piece of evidence that may be helpful in having faculty realize where they presently stand as educators or researchers as their yearly accomplishments are tracked, and new goals are devised for the next academic year. These mentor-mentee matches are another example of formation of CoP. Additionally, the act of mentoring allows individuals to connect with their personal roots and values, to be caring and collaborative ( Thibault, 2019). How one perceives their own abilities and their human experience is an important element in Medical Humanism, with self-directed professional learning processes being a primary modality of self-efficacy and creative thinking outlets ( Thibault, 2019), and thus having mentors guide mentees on their professional path is an opportunity to capture this.

Recognizing the diverse needs of our faculty, and the importance of having role models for underrepresented minorities and ethnicities, we launched two special interest groups, the Junior Faculty Interest Group (JFIG) and the Women in Medicine group. The JFIG is geared for faculty who are Assistant Professors and with less than 7 years of experience, as a networking group to share resources on going through the academic promotion process, how to find mentorship for support, highlights of buzz topics in academic medicine, and general comradery among faculty in a similar career stage, a less formal but still deliberate opportunity to join a CoP. The group hosts early morning and noontime faculty development sessions with internal and external speakers. The Women in Medicine group was formed as a platform to host topics most pertinent to our faculty identifying as women. The group has hosted workshops focusing on imposter syndrome, negotiation skills, work-life integration, and mastering time. Additionally, the Women in Medicine group has a particular focus on the number of academic promotions and leadership positions held by women faculty at our institution, as women in medicine are underrepresented in leadership and management roles in many academic medical centers, despite some progress over the last three decades ( Yoo *et al.*, 2021).

***Faculty coaching program*.** Distinguishing from mentoring, our coaching program was launched in 2023 and is available to faculty who require time-limited, task-oriented guidance in either two areas: building and sustaining an educator portfolio, or writing for publication. We have 14 mid to late career faculty who voluntarily serve as coaches to give 1:1 sessions on building educator portfolios and publishing scholarly papers, two areas that our faculty often verbalize the desire for guidance on in their path to academic promotion. This individualized development approach to faculty coaching has a dual-outcome with respect to faculty PIF. For those faculty seeking out coaches, they are able to gain direction tailored to concrete products they will need to finish related to education and research for their own development. Concurrently, the faculty coaches are able to exercise their knowledge and expertise in either education or scholarship, affording them a contribution to their own character as leaders in these areas. Coaching conversations enable learners’ development to promote PIF ( Parsons *et al.*, 2021). This continuation of self-directed learning allows for further individual differentiation of faculty professional identity reflection, for both coach-ee and coach ( Cruess *et al.*, 2015).

***Faculty council*.** The Faculty Council is the official representative body of our faculty at the school of medicine, established to promote open and frank discussion of issues concerning shared goals of excellence in medical education, clinical care, and scientific research, with special emphasis on supporting the school’s mission of promoting excellence in primary care fields of medicine. This council was founded as the medical school was formed and was created to represent and advocate for the faculty, in support of the academic mission of the medical school. The Council focuses on a variety of topics and issues pertaining to faculty and overall professional development, including promotions and tenure, wellbeing, and planning related to medical education. Monthly council meetings are open to all faculty, but information is also disseminated by email, the Council’s website, and communications from the elected department representatives.

 The Council also runs the elections for school of medicine-wide committees, including student progress/promotions, diversity and inclusion, grievance, curriculum, admissions executive committee, and appointment, promotions, and tenure. Each of these committees has both appointed and elected members, and the Council assists the Dean’s Office by holding the elections.

We designed our Faculty Council with the tenets of PIF in mind to support our faculty in many ways. By serving on the Council, and/or joining in events or programs, there are several opportunities for faculty engagement. These venues allow for reflection, increased self-awareness, and improved understanding of what aspects of faculty development and wellbeing might be of greatest interest and impact to our faculty, in order to build CoP through social and professional networks and cultivate leadership development ( Steinert *et al.*, 2019).

Faculty members who have an interest in getting involved in the Council can choose to do so in one or more of a variety of ways. First, all faculty can benefit from the Council’s activities and its work to disseminate information about several resources and the overall infrastructure that exists to support the PIF and overall development of our faculty. Second, faculty can volunteer to serve on one of the Council’s standing committees, including academic affairs, benefits and communications, student life, elections, faculty, and planning, budget, and salary.

In addition to the monthly meetings, the Council plans and holds a variety of sessions and events that are designed to promote professional enrichment, networking, and wellness to cultivate PIF. The Council’s leadership and communications team also work with other groups and committees to help disseminate information about events and programs that may be of interest to the faculty. Some of the sessions are web-based, others are in person, and others are hybrid. Topics have included education and guidance about the academic promotions process, annual faculty appreciation and social events, clinical practice development, benefits updates, time management, and wellbeing. Additional peer support and wellness events are planned for the current year, as the Council assesses faculty needs formally at least annually and uses that information to plan for the coming year.

## Results

To date, we have had 75 Faculty Scholars program graduates that are still active at our institution. Graduates of both the Educator and Researcher pathways (and earlier graduates of the program before there were designated pathways) often return as Faculty Scholars program lecturers, coaches, and mentors in subsequent years, to maintain their relationship within the growing CoP of program alumni. The rich diversity of social relations and peer modeling available within the growing CoP is expected to increase satisfaction of CoP participants and improve the “stickiness” of their extended professional identities. Over half of our Faculty Scholars graduates now hold educational leadership roles at our institution, ranging from directors in undergraduate and graduate medical education, as well as one dean in our medical school.

In the past two years, we have ensured that faculty at the assistant professor level on the Educator and Scholar tracks have had an assigned mentor (87 faculty in 2022, 91 faculty in 2023, respectively). Similarly, in the past year and one quarter, the faculty coaching program has delivered 14 guided sessions on educator portfolios with faculty, and 19 directed sessions on scholarly writing.

Approximately 30 faculty members serve as elected members of Faculty Council committees each year, each serving 3–4-year terms, depending on the committee. Of note, this is in addition to more than 45 other faculty members who have been appointed by the Dean or her/his designee to serve on one or more of these committees. These opportunities have allowed a substantial number of faculty to explore and engage in a variety of meetings, committees, and events that can contribute to their Professional Identity Formation (PIF) relating to CoP and shared governance, increasing their sense of belonging while also helping them to identify areas of personal interest as well as improvement. Each year, more than 45 faculty members serve as standing committee members. Over the past 5 years, more than 130 different faculty members have served on one or more of these standing committees. Other faculty members serve as elected department representatives or executive officers of the Council, totaling another 25–30 faculty per year. As the institution and the medical school have grown, so have the size of many departments. Each spring, Council leadership assesses the size of each department, and then calculates the updated number of elected representatives per department for the next academic year. At the time of this manuscript, 31 faculty serve as elected department representatives, with many others serving as alternates. The size of the Council, based on the number of elected representatives, has grown by approximately 18% over the five years since the Faculty Council was founded.

## Conclusion

### Future implications for PIF in faculty development and faculty engagement

There is a need to build on the use of PIF in faculty development and engagement initiatives within academic medical centers. In contrast with the quantity of literature reporting in the use of PIF for students and trainees in medical education, there is much room to expand on what is published about PIF in faculty ( Mahajan *et al.*, 2022; McMains *et al.*, 2023; Mount *et al.*, 2022).

Many faculty enter our faculty development and engagement programs upon hire anticipating formalized educator and leader roles they can assume in either undergraduate or graduate medical education. Furthermore, faculty express aims to produce more scholarship in the form of peer-reviewed publications, and feel the need to acquire fundamental research skills to assist in their clinical investigations. By immersing themselves in our described programs, participants engage with a community of colleagues with similar interests and goals, and further explore “who they can become” in their careers. Through the lens of PIF, our faculty development and engagement initiatives allow for learning through peer-affiliations possible in CoP context, with the chance to view role models, give and receive feedback, and obtain direct instruction in a social-relational, as opposed to individualistic, environment. ( Cantillon *et al.*, 2019; Irby & Hamstra, 2016). A continual encounter with peers in CoP extends the premise of PIF further by not only addressing the educator, researcher, and leader identities of faculty when they enter these programs, but also by working on “who they can become” after program participation.

Through our experience in designing and implementing our faculty development and engagement initiatives, we suggest using a few factors to gauge success of the programs: faculty participation, faculty partnerships/collaborations, and emerging roles in education and leadership.

Professional identity is invaluable for the performance, career growth, satisfaction and retention of our physician workforce in academic health centers. By setting forth programmatic initiatives we have described above and drawing upon key characteristics we have outlined such as CoP, Medical Humanism, and URiM, a solid foundation and frame of reference can be built to assist faculty in academic health centers from which future studies in this area can be generated. The larger field of faculty development may also benefit from future studies examining PIF dynamics compared across health educators and other profession/vocation cohorts.

## Ethics and consent

Ethical approval and consent were not required

## Data Availability

No data associated with this article
