# Supplementary material for: Extending Professional Identity Formation to develop academic faculty for a new medical school
Source: MedEdPublish (2016). 2024 Jul 22;14:57. [Version 1] doi: 10.12688/mep.20510.1 (PMC11384206; doi:10.12688/mep.20510.1)
Supplement: Peer review history

# Reviewer report: Reviewer response for version 1

**DOI:** 10.21956/mep.21950.r38613

**Published:** 2024-09-09

**Authors:** Maria Blanco

**Affiliations:** 1 Tufts University School of Medicine, Boston, Massachusetts, USA

First, I want to thank you for the opportunity to review your scholarly work and share my thoughts on your paper. As a faculty developer in health sciences education for over twenty-four years, I read your paper with great interest. I believe that sharing our programs allows us to learn from one another's faculty development practices while advancing the field as a whole, and I see your paper as an important contribution to that effort. I commend you for this.

 While I can identify the theoretical frameworks that informed your program based on your description, I am still curious about the overall educational design. Specifically, I was hoping the paper would address the learners' needs, program goals, learning objectives, methods, and evaluation approach, and how these components align with the theoretical frameworks in a clear and straightforward manner. Although you describe the different methods and programs offered, I find it challenging to fully understand their cohesive relevance without a clearer description of the underlying educational design.

 For example, you could include a roadmap of your design, mapping the objectives of each offering along with their expected processes and outcomes. Additionally, incorporating more literature on faculty development related to evidence-based interventions and existing frameworks on educators' activities and educational scholarship would help better contextualize your program and interventions.

 Similarly, I was curious about your program evaluation framework and how it informed the results you report. Regarding your results, I was hoping the paper would discuss how they relate to the professional identity formation of medical educators, beyond simply describing participant engagement in numerical terms.

 In summary, for a process-focused paper like this, I would recommend bringing the programmatic design to the forefront. You might consider detailing the individual programs in separate papers, while here providing the objectives and highlights of each to better illustrate the programmatic and theoretical alignment.

 Thank you again for the opportunity to review your paper.

Is the rationale for developing the new method (or application) clearly explained?

Yes

Is the description of the method technically sound?

Yes

Are the conclusions about the method and its performance adequately supported by the findings presented in the article?

Partly

If any results are presented, are all the source data underlying the results available to ensure full reproducibility?

No

Are sufficient details provided to allow replication of the method development and its use by others?

No

Reviewer Expertise:

faculty development-mentoring- program evaluation-educational research and scholarship

I confirm that I have read this submission and believe that I have an appropriate level of expertise to confirm that it is of an acceptable scientific standard, however I have significant reservations, as outlined above.

# Reviewer report: Reviewer response for version 1

**DOI:** 10.21956/mep.21950.r38611

**Published:** 2024-09-09

**Authors:** Hugh Stoddard

**Affiliations:** 1 Huffington Department of Education, Innovation, and Technology, Baylor College of Medicine School of Medicine, Houston, Texas, USA

Review of Extending Professional Identity Formation to develop academic faculty for a new medical school [version 1]

 I appreciate the opportunity to review this interesting and well-written manuscript.  The overall intent is clear and is a refreshing addition to the copious literature on faculty development in academic health centers.  Following are several thoughts/questions for the authors to consider:

 1) The general tenor of this manuscript feels like a ‘tweener’ – somewhere between a quantitative mindset and a qualitative mindset.  On one hand, the authors are concerned about generalizability of their PIF-based faculty development initiative to other institutions (e.g. “academic medical centers can draw upon the formation of these programs to best serve their faculty”), yet on the other hand, they eschew quantification and structured methods in favor of a rich description of the phenomenon of interest (as noted in their response to a previous reviewers comments #4 & #5).  For current purposes, I suggest thinking more qualitatively; perhaps revising this manuscript to read like a case study would make it more easily digestible to readers.  Granted, there are already descriptions of several initiatives, but the narratives weren’t as insightful as I had hoped, given my interest in the PIF framework that the authors adopted.

 2) In healthcare professions, PIF moves towards, “a developed stage when the external values of the profession become internal values of the individual.”  Yet, it’s not clear to me what that ‘developed stage’ for a healthcare faculty member might look like.  For medicine, the ideals and identity of the profession have undergone discussion for generations – appropriately so.  My experience in the field is that we don’t have much evidence that there is a shared mental model of characteristics for ‘healthcare faculty’ as a unique profession.

 3) In general, this manuscript represents a fascinating new line of inquiry and innovation.  I look forward to reading revisions of this manuscript and learning more from this author group!

Is the rationale for developing the new method (or application) clearly explained?

Yes

Is the description of the method technically sound?

Partly

Are the conclusions about the method and its performance adequately supported by the findings presented in the article?

Yes

If any results are presented, are all the source data underlying the results available to ensure full reproducibility?

Not applicable

Are sufficient details provided to allow replication of the method development and its use by others?

Partly

Reviewer Expertise:

Medical education curriculum and assessment

I confirm that I have read this submission and believe that I have an appropriate level of expertise to confirm that it is of an acceptable scientific standard.

# Reviewer report: Reviewer response for version 1

**DOI:** 10.21956/mep.21950.r38618

**Published:** 2024-09-09

**Authors:** Steve Taff

**Affiliations:** 1 Washington University in St Louis, St. Louis, Missouri, USA

This is an timely, interesting, and well-written manuscript that further highlights the often subtle (but very important) role of faculty development activities in the ongoing process of professional identity formation. A few comments for the authors to consider include:

 1. In the introduction, please consider adding a short discussion of the needs assessment process/data that identified the gaps that these new programs address.

 2. A table with key/representative learning objectives for each program would enhance the description via a visual. 

 3. Since this is a paper describing program development, it is expected that no assessment data would be collected (yet). That said, I think adding just a bit of discussion about which metrics/outcomes are of priority interest and how you plan on evaluating the programs would be a helpful addition. Perhaps use Kirkpatrick's Training Model to frame this.

 Thank you for the opportunity to review this manuscript, I enjoyed the read!

Is the rationale for developing the new method (or application) clearly explained?

Yes

Is the description of the method technically sound?

Yes

Are the conclusions about the method and its performance adequately supported by the findings presented in the article?

Yes

If any results are presented, are all the source data underlying the results available to ensure full reproducibility?

Partly

Are sufficient details provided to allow replication of the method development and its use by others?

Partly

Reviewer Expertise:

Qualitative research, curriculum design, faculty development, leadership, scholarship of teaching & learning, faculty and learner belonging and wellbeing.

I confirm that I have read this submission and believe that I have an appropriate level of expertise to confirm that it is of an acceptable scientific standard.

# Reviewer report: Reviewer response for version 1

**DOI:** 10.21956/mep.21950.r38125

**Published:** 2024-08-22

**Authors:** Nicole Rockich-Winston

**Affiliations:** 1 Pharmacology and Toxicology Department, Medical College of Georgia, Augusta University, Augusta, GA, USA

Thank you for the opportunity to review this manuscript. The authors describe approaches to developing professional identity formation (PIF) within faculty development initiatives. The authors offer interesting programs that could be instituted elsewhere for PIF development for academic medicine faculty. 

 Areas recommended for improvement include:

 (1) Clearly defining and describing key terms and methods in the Introduction. For example, what do you mean by social-contextual approaches? Also, what has been done already with respect to PIF in faculty development, beyond academic medicine? Why is it important to develop PIF in academic clinicians?

 (2) Please include racial/ethnic demographic information in your description of faculty and for those who completed the program. This will help with evaluating whether department chairs are focusing efforts on underrepresented groups in medicine.

 (3) As written, the programs would be difficulty to replicate elsewhere because each program lacks a detailed, step-by-step description of what occurs in these programs. Maybe adding a table with more information would help.

 (4) The methods section needs a clear description of what and how you measured the success of your programs. Then, this would be reflected in the results section. 

 (5) Because the data collection is not defined in the methods section, the results section seems a bit disorganized. Once the methods are clearly stated, the results section can be organized around this. Additionally, a table would be helpful in summarizing results.

 (6) The discussion/conclusion should include a thorough summary of PIF faculty development programs in the literature both inside and outside of medicine.

Is the rationale for developing the new method (or application) clearly explained?

Yes

Is the description of the method technically sound?

Partly

Are the conclusions about the method and its performance adequately supported by the findings presented in the article?

Partly

If any results are presented, are all the source data underlying the results available to ensure full reproducibility?

Partly

Are sufficient details provided to allow replication of the method development and its use by others?

No

Reviewer Expertise:

Mixed method studies, professional identity formation

I confirm that I have read this submission and believe that I have an appropriate level of expertise to state that I do not consider it to be of an acceptable scientific standard, for reasons outlined above.

# Response

**Published:** 2024-08-22

**Authors:** Jeannine Nonaillada

**Affiliations:** NYU Grossman Long Island School of Medicine, USA

Thank you for taking the time to review. Here is our response:

1. **Reviewer's comment: **Clearly defining and describing key terms and methods in the Introduction. For example, what do you mean by social-contextual approaches? Also, what has been done already with respect to PIF in faculty development, beyond academic medicine? Why is it important to develop PIF in academic clinicians?  
**Response:** To this point, we direct the reviewer to the Introduction. “Academic health centers have a responsibility to foster professional development approaches and engagement environments for faculty to elevate both knowledge and sense of belonging as medical educators.”  
  We specifically outline that through the lens of PIF, social-contextual approaches are “sharing in communal learning and training, as well as networking with others”.  
2. **Reviewer's comment:** Please include racial/ethnic demographic information in your description of faculty and for those who completed the program. This will help with evaluating whether department chairs are focusing efforts on underrepresented groups in medicine.        
**Response:** While we certainly could include racial/ethnic demographic information about the overall description of our faculty as a whole, this paper is not about one single program and but programmatic development of many initiatives with the underlying framework of PIF. Thus, unclear which aspect you are referring to when you say “completed the program”.
3. **Reviewer's comment:** As written, the programs would be difficulty to replicate elsewhere because each program lacks a detailed, step-by-step description of what occurs in these programs. Maybe adding a table with more information would help.
**Response: **While most academic health centers, already have faculty development, faculty mentoring and faculty coaching programs, and there is a wealth of literature on such programs already, this paper is to describe how we applied the underlying framework of PIF for our institution as a whole, while providing examples of our faculty programs. And thus, the focus is on the theoretical application, which we provide the connections to PIF throughout the paper for each aspect of our programming.
4. **Reviewer's comment:** The methods section needs a clear description of what and how you measured the success of your programs. Then, this would be reflected in the results section.
5. **Reviewer's comment:** Because the data collection is not defined in the methods section, the results section seems a bit disorganized. Once the methods are clearly stated, the results section can be organized around this. Additionally, a table would be helpful in summarizing results.
**Response: **To points 4 and 5, as this is not a traditional research paper, but a paper describing the application of a theoretical framework to programmatic development, success of our programs was measured as we state in the paper as *faculty participation, faculty partnerships/collaborations, and emerging roles in education and leadership. *There is no “pre and post” data as in a traditional research paper.
6. **Reviewer's comment: **The discussion/conclusion should include a thorough summary of PIF faculty development programs in the literature both inside and outside of medicine.
**Response: **As we stated in the introduction, the focus of this paper is on academic medical centers, and thus that is where we centralized the literature and the discussion on that. We state early on in the paper that PIF is not as widely reported on for faculty, but more for medical students, and thus we share our experience through this theoretical application paper to shed light on how other institutions may apply this framework to their own existing programming and/or new programming.
